# Lysophosphatidic Acid Signaling in Obesity and Insulin Resistance

**DOI:** 10.3390/nu10040399

**Published:** 2018-03-23

**Authors:** Kenneth D’Souza, Geena V. Paramel, Petra C. Kienesberger

**Affiliations:** Department of Biochemistry and Molecular Biology, Faculty of Medicine, Dalhousie University, Dalhousie Medicine New Brunswick, Saint John, NB, E2L 4L5 Canada; kn900230@dal.ca (K.D.); gn240575@dal.ca (G.V.P.)

**Keywords:** lysophosphatidic acid, lysophospholipids, autotaxin, obesity, insulin resistance, adipocytes, cardiovascular disease, diet

## Abstract

Although simple in structure, lysophosphatidic acid (LPA) is a potent bioactive lipid that profoundly influences cellular signaling and function upon binding to G protein-coupled receptors (LPA1-6). The majority of circulating LPA is produced by the secreted enzyme autotaxin (ATX). Alterations in LPA signaling, in conjunction with changes in autotaxin (ATX) expression and activity, have been implicated in metabolic and inflammatory disorders including obesity, insulin resistance, and cardiovascular disease. This review summarizes our current understanding of the sources and metabolism of LPA with focus on the influence of diet on circulating LPA. Furthermore, we explore how the ATX-LPA pathway impacts obesity and obesity-associated disorders, including impaired glucose homeostasis, insulin resistance, and cardiovascular disease.

## 1. Introduction

Apart from serving as integral constituents of cellular membranes and sources of energy, lipids can play an important role as signaling molecules. These so-called ‘bioactive lipids’ initiate cell signaling cascades intracellularly (e.g., phosphatidylinositol (PI) and diacylglycerol (DAG)) [1,2] and/or extracellularly (e.g., sphingosine-1-phosphate (S1P), saturated fatty acids) [3,4,5] to influence a plethora of biological processes including cell proliferation, inflammation, survival, and development. The list of bioactive lipids is constantly expanding with new species being identified, in part through advances in lipidomic analyses [6].

**Figure 1:**
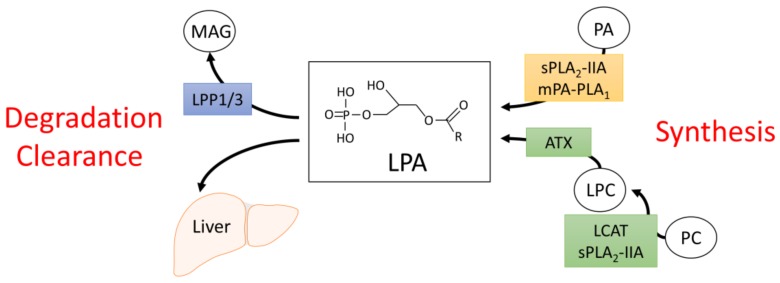
Metabolism of circulating lysophosphatidic acid (LPA). LPA is either synthesized from phosphatidic acid (PA) through the actions of PLA_1_/PLA_2_ or via autotaxin (ATX)-mediated hydrolysis of lysophosphatidylcholine (LPC). Clearance of circulating LPA involves its rapid degradation to monoacylglycerol (MAG) through the actions of LPP1/3 or hepatic uptake of LPA. sPLA_2_-IIA, group IIA secretory phospholipase A_2_; mPA-PLA_1_, membrane-bound PA-selective phospholipase A_1_; LCAT, lecithin–cholesterol acyltransferase.

An extensively studied bioactive lipid is lysophosphatidic acid (LPA), the simplest of all glycerophospholipids, which contains an acyl moiety esterified to the glycerol-3-phosphate backbone (Figure 1). LPA is a very potent signaling molecule, capable of activating a variety of signaling pathways via binding to six identified LPA receptors (LPA1-6) [7]. LPA is found in virtually all biological fluids, where its concentration can vary by orders of magnitude. For example, in rat cerebrospinal fluid, LPA concentration is as low as 40 fM [8], whereas in blood, LPA can range from 0.1 µM in plasma and up to 10 µM in serum [7]. However, it is important to note that locally produced LPA likely reaches much higher concentrations than circulating LPA [9]. The importance of LPA in disease has been underscored by studies linking aberrant LPA signaling to a broad range of pathophysiological conditions, including cancer, arthritis, pulmonary fibrosis, neurological disorders, and obesity-induced insulin resistance and impaired glucose homeostasis [7,10,11,12,13,14,15].

Under physiological conditions, LPA influences diverse cellular and organismal processes, including proliferation and growth, survival, development, chemotaxis, vasoregulation, and calcium dynamics [7]. The diversity of cellular responses to LPA is likely mediated by distinct LPA receptor tissue expression patterns, receptor–ligand kinetics, and substrate/acyl chain specificity of LPA receptors. For example, while all six LPA receptors are expressed in the murine and human heart and cardiomyocytes, LPA3 mRNA is undetectable in murine cardiomyocytes and human subcutaneous adipose tissue [16]. However, a comprehensive understanding of LPA receptor expression at baseline and under pathophysiological conditions is still lacking in many tissues. This is exemplified by a study showing the presence of LPA1, 3, and 4 in mouse skeletal muscle, while it remains unclear whether LPA5 and 6 are present in this tissue [17].

In addition to their potential tissue-specific expression, LPA receptors also show differences in receptor–ligand kinetics. LPA1, 2, 4, and 5 have an apparent K_d_ for 1-oleoyl-LPA (18:1 LPA) between 45 and 100 nM, whereas LPA3 has a K_d_ of 206 nM [18,19,20]. LPA6 failed to show specific binding to 1-oleoyl-LPA; this could be due to low affinity of LPA6 for this LPA species or due to rapid off rates of LPA [20]. To date, only limited information is available on potential differences in substrate specificities of LPA receptors. For example, 2-acyl-LPA is a more potent ligand for LPA6 than corresponding 1-acyl-LPA species [20]. When varying the acyl chains at the *sn*-1 position, LPA6 prefers linoleoyl (18:2) and oleoyl (18:1) containing LPA over saturated (myristoyl (14:0), palmitoyl (16:0), stearoyl (18:0)) and polyunsaturated (arachidonoyl (20:4)) LPA species [20]. LPA3 also prefers 2-acyl-LPA species; however, only unsaturated LPAs appear to be ligands for this receptor [18,20,21]. LPA1 and 2 do not show preference for either 1- or 2-acyl LPA and have broad acyl chain specificities, except for lauroyl-LPA (12:0 LPA) [21]. LPA5 is unique in that it prefers LPA species with ether-linked radyl chains, rather than ester-linked species [22,23]. With regard to acyl chain specificity, longer saturated 1-acyl LPA species (18:0 and 20:0) are poorer ligands for LPA5 compared to 16:0 LPA and unsaturated species (18:1, 18:2, 18:3, and 20:4 LPA) [23]. Dynamic changes in LPA species in different biological fluids could favor the activation of specific LPA receptors. For example, in mouse plasma, the major LPA species are ester-linked and contain 16:0, 18:0, 18:1, 18:2, and 20:4 acyl chains, at concentrations ranging from 10–100 nM [24]. A minor fraction of plasma LPA is ether-linked (<10 nM) [24]. Increases in ether-linked LPA, such as during platelet activation, could function to selectively activate LPA5 [23]. Currently, the distribution of 1- versus 2-acyl LPA species in biological fluids is unknown; future studies should aim to discriminate between these LPA species and examine their role in physiological and pathological processes.

## 2. Synthesis and Degradation of LPA 

Due to its potent bioactive nature, LPA levels are tightly regulated. Circulating LPA can be generated through two distinct enzymatic mechanisms (Figure 1). In the first, phosphatidic acid (PA) is converted to LPA through the actions of group IIA secretory phospholipase A_2_ (sPLA_2_-IIA) or membrane-bound PA-selective PLA_1_ (mPA-PLA_1_) [25,26]. PA itself can be presented at the cell surface through phospholipid scrambling or generated extracellularly via phospholipase D-mediated cleavage of phosphatidylcholine (PC) [25,26]. The second major mechanism of LPA synthesis involves the conversion of PC to lysophosphatidylcholine (LPC) through the actions of lecithin–cholesterol acyltransferase (LCAT) or sPLA_2_-IIA [26]. LPC can then be hydrolyzed to form LPA through the activity of the lysophospholipase D, ectonucleotide pyrophosphatase/phosphodiesterase 2 (ENPP2), an enzyme more commonly referred to as autotaxin (ATX) [27,28].

Several lines of evidence suggest that the ATX-mediated mechanism of LPA synthesis produces the majority of extracellular LPA in vivo. LPA formation is significantly restricted in plasma samples of LCAT-deficient patients [26]. More strikingly, LPA levels closely correlate with ATX protein content and/or activity. Heterozygous whole-body ATX-knockout (ATX^+/−^) and fat-specific ATX-knockout (FATX^−/−^) mice have approximately 50% and 40% less circulating LPA than wild type mice, respectively [27,29,30,31,32]. Inducible whole-body deletion of ATX in adult mice, resulting in ~80% reduction of ATX mRNA levels and plasma ATX activity, was similarly associated with a ~60% decrease in plasma LPA [33]. Pharmacological inhibition of ATX, through the use of potent inhibitors such as PF-8380, led to a >95% decrease in plasma LPA [33,34]. Conversely, overexpression of ATX in mice corresponded with increased circulating LPA levels [29,31,35].

The major route for LPA degradation involves its dephosphorylation to monoacylglycerol (MAG) through mammalian lipid phosphate phosphatases (LPP) (Figure 1) [36,37]. There are three enzymes characterized in this family: LPP1 (PPAP2A), LPP2 (PPAP2C), and LPP3 (PPAP2B) [38]. Knockdown of LPP1 in mice increases circulating LPA levels and extends the half-life of injected LPA 4-fold, with no obvious effect on phenotype [39]. In contrast to LLP1 deficient animals, LPP3 knockout mice are not viable, due to their inability to form a chorioallantoic placenta and vascular defects in the yok sac [40]. Culture of embryonic mouse fibroblasts lacking LPP3, however, resulted in a ~2.5-fold increase in extracellular LPA [40]. LPP2 mice are viable with no overt phenotype, although levels of LPA were not reported [41]. LPP2 may play a specific role in regulating the timing of cell cycle progression, as increasing LPP2 activity in fibroblasts leads to the premature entry of cells into the S-phase of the cell cycle and decreases proliferation rate [42,43]. LPP2 does not influence levels of LPA in these fibroblast models [33], suggesting that the effects of LPP2 modulation on cell cycle progression are not due to changes in LPA degradation. Taken together, these findings suggest that LPPs may play unique, isoform specific roles in regulating circulating LPA levels during development and postnatal life. Apart from the degradation of LPA through LPPs, LPA is also cleared from circulation by nonparenchymal cells in the liver [24]. Intravenously administered LPA rapidly accumulates in the mouse liver and ligation of the hepatic circulation blocks the clearance of LPA, suggesting that uptake of LPA by liver cells is an important mechanism for the regulation of circulating LPA levels and contributes significantly to the short half-life (<30 s) of LPA in the blood stream [24].

### 2.1. Sources of Circulating LPA

**Figure 2:**
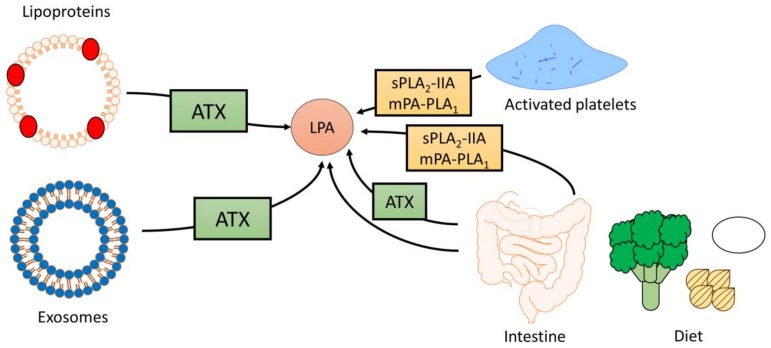
Sources of circulating LPA. LPA can be generated from a variety of sources, including lipoproteins, exosomes, activated platelets, and diet.

LPA is primarily bound to serum albumin in the blood stream with reported concentrations of up to 1 μM in plasma and >10 μM in serum, demonstrating that LPA levels can vary greatly dependent on factors such as nutritional status and clotting (Figure 2) [7,44]. During clotting, platelets produce a significant amount of LPA. This is exemplified by a study showing that administration of an antiplatelet antibody in rats reduces serum LPA levels by almost 50% [26]. Similarly, pharmacological inhibition of platelet aggregation using an integrin αIIbβ3 antagonist reduces circulating LPA levels by 70% in a mouse model of metastatic breast cancer [45]. In addition to platelets, circulating lipoproteins serve as a source of LPA, particularly when subjected to oxidation. For example, the production of LPA from oxidatively modified low-density lipoproteins (oxLPL) via ATX is critically required for monocyte recruitment and promotion of atherosclerosis [46]. Moreover, ATX activity is associated with lipoprotein (a) [47], which transports oxidized phospholipids and LPC generated by lipoprotein-associated PLA_2_ [48], suggesting that lipoprotein (a) also constitutes a source of LPA. Interestingly, a recent study demonstrated that exosomes may serve as a vehicle and/or delivery system for ATX-LPA (Figure 2) [49]. Packaging of LPA in exosomes may be a means by which LPA is delivered to target tissues and cells for signaling, and could potentially increase the stability of circulating LPA. It remains to be determined whether LPA present in exosomes significantly contributes to circulating LPA levels in vivo and whether the concentration of LPA in exosomes changes during disease states. Moreover, future studies should investigate whether LPA derived from different sources varies in fatty acid composition and signaling properties. It should be noted here that serum is an unsuitable source for the determination of circulating LPA since platelet activation during clotting can significantly increase LPA levels, thereby leading to the overestimation of blood LPA levels. Therefore, blood LPA measurements should be conducted using plasma collected in tubes containing an anticoagulant such as EDTA [7,26,50,51]. Additionally, the use of siliconized tubes for plasma collection should minimize LPA binding to the tube surface [33,52].

### 2.2. The Influence of Diet on LPA

Marked changes in ATX-LPA levels are associated with many physiological and pathophysiological processes, including development, cell differentiation, cancer, atherosclerosis, and myocardial infarction, and have also been linked to alterations in the nutritional status [10,32,53,54]. Circulating LPA, along with ATX, are regulated by feeding–fasting, with higher levels of ATX-LPA detected in the fed versus fasted state in animal models [55,56]. In addition to the acute nutritional regulation of ATX-LPA, several studies show that chronic overfeeding of animal models with obesogenic diets results in altered circulating LPA levels. Feeding male FVB mice a high fat–high sucrose (HFHS, 45% kcal fat, 17% kcal sucrose) diet for 13 weeks elevates plasma LPA levels by 62% [30]. Increases in plasma LPA were also noted in male C57Bl6/J mice fed a HFHS diet or high-fat diet (60% kcal fat, no added sucrose) for a shorter period of nine and eight weeks, respectively [57,58]. Similarly, female low-density lipoprotein receptor-null (LDLR^−/−^) mice exhibit increased unsaturated, but not saturated levels of LPA in the small intestine following consumption of a Western diet (42% kcal fat, 34% *w*/*w* sucrose, 0.2% *w*/*w* cholesterol) for only three weeks, which is also paralleled by an increase in 20:4 LPA in plasma [59]. Since LPA content in the Western diet is lower than in the control chow diet and animals were fed the same amount (by weight) of the diet, increased intestinal LPA in mice fed a Western diet is unlikely the result of higher consumption of preformed LPA [59]. Taken together, these studies suggest that the consumption of a fat-rich diet leads to increased circulating levels of LPA in mice of different genetic backgrounds. While reports on LPA measurements in humans are limited, a very recent study showed that plasma LPA positively correlates with body mass index (BMI) [51], an indicator of nutritional imbalance. The same study also suggests that fasting has a marginal effect on circulating LPA concentrations in humans, although these data have not been adjusted for sex—LPA levels are higher in women compared to men, and the exact duration of fasting is unclear [51].

Although the precise mechanisms underlying the dietary regulation of LPA levels remain to be uncovered, it is possible that higher dietary content of preformed LPA contributes to variations in circulating LPA levels. LPA has been detected in several plant and animal foods, including eggs [60], cabbage leaves, broccoli [61], soybeans, and sunflower seeds (Figure 2) [62]. Dietary LPAs, especially those containing mono- and polyunsaturated fatty acids, appear to be well-absorbed in the mouse and rat intestine [59,63]. Interestingly, however, a standard chow diet contains higher levels of preformed LPA than a Western diet, suggesting that increases in circulating and intestinal LPA in LDLR^−/−^ mice following Western diet feeding are not due to increased absorption of preformed LPA [59]. Therefore, it is plausible that obesogenic, lipid-rich diets increase LPA levels in vivo via a more indirect mechanism, e.g., by influencing levels of LPA precursor lipids (see above). For example, PA can be converted to LPA by pancreatic phospholipase A_2_-mediated hydrolysis [61,64]. Since levels of preformed PA were much lower than levels of intestinal LPA in Western diet-fed LDLR^−/−^ mice, this mechanism does not appear to significantly contribute to increased LPA content in this mouse model either [59]. However, compared to chow-fed mice, Western diet-fed LDLR^−/−^ mice showed an 8- and 10-fold increase in intestinal and plasma LPC content, respectively [65], although preformed LPC or PC levels were similar or lower in the Western versus chow diet [59,65]. This suggests that increases in LPC content may underlie the Western diet-induced upregulation of LPA. Within the enterocyte, LPC can be converted to LPA via ATX-mediated hydrolysis [53]. Interestingly, inhibition of ATX using PF-8380 only significantly decreases levels of unsaturated LPA in the jejunum, liver, and plasma of male LDLR^−/−^ mice fed a chow diet supplemented with oleoyl-LPC (18:1 LPC), suggesting that saturated LPA is formed by an ATX-independent mechanism in the intestine [53].

The third, and perhaps most prominent mechanism by which diet can modulate LPA levels is through the upregulation of ATX. Prior studies using mice with high-fat diet-induced obesity show increased ATX mRNA and protein expression in adipose tissue, a major source of circulating ATX [30,58]; this is also reflected by increased circulating ATX and/or serum ATX activity in obese mice, which correlates well with increases in LPA [55,58]. On the other hand, a study by Nishimura et al. [31] shows that an obesogenic diet decreases ATX levels in adipose tissue and circulation. The reason for this discrepancy between studies is not immediately clear, since only minor differences in experimental conditions are evident. Therefore, future studies need to clarify precisely how diet-induced obesity is linked to changes in ATX-LPA.

## 3. ATX-LPA Signaling in Obesity

In humans, the relationship between ATX-LPA and obesity also remains somewhat unclear. In severely obese women (BMI 35.0–64.5), serum ATX does not correlate with markers of obesity, including weight, BMI, or waist circumference [66]. However, ATX mRNA is significantly increased in the visceral adipose tissue of massively obese female patients (BMI > 40.0) compared to non-obese controls (BMI < 25.0) [67]. Moreover, serum ATX correlates with both BMI and waist circumference in older overweight or obese patients (BMI: 25.0–37 kg/m^2^) [68]. Consistent with these data, 16:0 LPA is significantly increased in obese (BMI > 30.0) individuals compared to individuals with normal BMI (BMI 18.5–25.0) [69]. On the other hand, a different study shows that ATX levels in subcutaneous adipose tissue and serum negatively correlate with BMI, respectively [31]. Notably, these data are not normalized to sex and the study population consists almost exclusively of individuals with normal BMI or preobesity, with less than 1% individuals being obese based on BMI [31]. Overall, evidence to date suggests that tissue ATX expression and circulating ATX-LPA levels may not correlate well with parameters of obesity across different study populations. Our understanding of the relationship between the ATX-LPA pathway and obesity can be improved by examining circulating LPA levels in human cohorts, in addition to ATX expression and activity. Brown et al. [16] demonstrated that mRNA levels of distinct LPA receptors in insulin sensitive mouse and human tissues are associated with obesity. For example, LPA4, LPA5, and/or LPA6 are significantly increased in myocardial tissue and cells from HFHS-fed mice and humans with preobesity or obesity [16]. These data suggest that changes in tissue LPA receptor expression may also contribute to alterations in ATX-LPA signaling during obesity.

### 3.1. Role of the ATX–LPA Axis in Preadipocyte Proliferation and Differentiation

Adipocyte hyperplasia and hypertrophy are two mechanisms by which adipose tissue expands during development and obesity [70]. Through autocrine and paracrine signaling, the ATX–LPA axis is believed to influence both processes and play a key role in altering adipose tissue biology and metabolism during obesity. The effect of ATX-LPA signaling on adipose tissue was examined predominantly using preadipocyte models (Table 1). Preadipocytes secrete low levels of ATX into the extracellular medium, which, in the presence of LPC, results in the production of minimal levels of LPA [28]. Nevertheless, even low concentrations of ATX-LPA stimulate preadipocyte proliferation, as was assessed in murine 3T3-L1 and 3T3-F442A preadipocytes and primary Pref1^+^ CD34^+^ adipocyte progenitors exposed to exogeneous ATX or LPA, consistent with the well-known mitogenic effect of LPA [31,71,72,73]. In agreement with these observations, mice with adipose-specific ATX deficiency have significantly fewer preadipocytes in the stromal vascular fraction of epididymal fat pads, suggesting that ATX-LPA signaling stimulates preadipocyte proliferation in vivo [31]. ATX-LPA-induced proliferation of white preadipocytes appears to be primarily mediated by LPA1, a major LPA receptor in Pref1^+^ CD34^+^ adipocyte progenitors, and possibly ras-mitogen activated protein kinase (MAPK) [31,71,73,74]. Interestingly, although knockdown of LPA1 diminishes preadipocyte proliferation induced by LPA, it only results in partial reduction of ATX-induced proliferation, suggesting that ATX can promote preadipocyte proliferation independent of LPA-LPA1 signaling [31]. Contrary to the ATX-LPA-induced proliferation of white preadipocytes, neither LPA nor ATX inhibitors appear to influence the proliferation of primary murine brown preadipocytes [75], suggesting that the ATX-LPA pathway stimulates preadipocyte proliferation specifically in white preadipocytes.

While overwhelming evidence points towards a pro-proliferative effect of LPA in white preadipocytes, studies examining the role of LPA signaling in preadipocyte differentiation produced more ambiguous results (Table 1). Notably, levels of ATX mRNA and secreted ATX protein and activity increase markedly during differentiation in 3T3-L1, 3T3-F442A, and primary preadipocytes, indicating a prominent role of ATX-LPA signaling in preadipocyte differentiation [28,31,55]. Indeed, some studies suggest that LPA is a potent suppressor of preadipocyte differentiation. Murine 3T3-F442A and 3T3-L1 preadipocytes, porcine DFAT-P preadipocytes, human Simpson–Golabi–Behmel syndrome (SGBS) preadipocytes, and primary murine white and brown preadipocytes do not differentiate into mature adipocytes as efficiently when incubated with LPA, as determined by the expression of adipogenic and lipid metabolism markers and lipid droplet/triacylglycerol accumulation [73,75,76]. Conversely, inhibition of ATX activity promotes the differentiation of primary murine brown preadipocytes [75]. The differentiation-inhibiting effects of ATX-LPA appear to be mediated through the LPA1-dependant downregulation of peroxisome proliferator-activated receptor γ2 (PPARγ2) in preadipocytes [73,76]. ATX-LPA-induced downregulation of PPARγ and PPARγ-sensitive proteins is also observed in mature 3T3-L1 adipocytes [55]. In agreement with this notion, the antiadipogenic effect of LPA is not observed in preadipocytes isolated from LPA1-knockout mice, which may underlie the increased adiposity in these mice [76]. However, a recent study showed that Pref1^+^ CD34^+^ preadipocytes isolated from epididymal white adipose tissue from mice with global heterozygous ATX deficiency differentiate less efficiently than preadipocytes from wild type mice, suggesting that the ATX-LPA pathway promotes preadipocyte differentiation (Table 1) [31]. It is possible that constitutive ATX deficiency and reduced LPA levels during and after development alter the adipogenic potential of preadipocytes in vivo. Indeed, the expression of adipogenic genes is reduced in preadipocytes from ATX^+/−^ and FATX^−/−^ mice [31], which may explain their impaired ability to differentiate.

### 3.2. Role of ATX-LPA in Diet-Induced Obesity

Despite the prominent role of ATX-LPA signaling in preadipocyte proliferation and differentiation demonstrated in vitro, modulation of the ATX-LPA pathway in vivo appears to have little effect on adiposity in mice at baseline [30,31,75,76]. Having said that, the profound impact of ATX-LPA signaling on adiposity becomes evident when mice are fed an obesogenic diet (Table 1). For example, administration of the LPA1/3 antagonist, Ki16425, for six weeks increases fat mass and the size of white adipocytes in HFHS-fed C57Bl6 mice [57]. The same group also showed that adipose-specific ATX deletion increases white and brown adipose tissue mass in HFHS-fed mice (on a mixed FVB/Bl6 background), which is associated with upregulated mRNA levels of PPARγ and PPARγ-sensitive genes, including adiponectin and leptin, predominantly in subcutaneous adipose tissue from these mice [30]. Conversely, a more recent study shows that global heterozygous and adipose-specific ATX deficiencies protect mice on a C57Bl6 background from diet-induced obesity, while adipose-specific ATX overexpression driven from the FABP4 promoter enhances adiposity following high-fat diet feeding [31]. The resistance to diet-induced obesity in FATX^−/−^ mice was ascribed to improved BAT function, lipid oxidation capacity, and energy expenditure [31]. Similarly, overexpression of ATX driven by the α1-antitrypsin promoter in FVB/N mice, resulting in a moderate increase in circulating ATX and LPA levels in adult mice, increases weight gain and adiposity following consumption of a HFHS diet [75]. This effect was linked to reduced expression of BAT-related genes, indicative of lower brown adipocyte abundance, in peripheral white adipose tissue of HFHS-fed ATX transgenic mice [75]. Differences in the background strain of mice and composition of obesogenic diets may have contributed to these, in part, divergent results among studies examining the role of ATX-LPA in diet-induced obesity [30,31,57,75]. Clearly, more research is needed to address the precise role of ATX-LPA signaling in diet-induced obesity. Future studies should employ ATX/LPA receptor inhibition/deletion in adult mice before and after the induction of obesity, to determine whether the ATX-LPA pathway impacts adiposity independent of its possible effect on preadipocyte development/programming and whether increased adiposity can be reversed or ameliorated by ATX-LPA modulation.

## 4. Relationship between ATX-LPA and Insulin Signaling/Resistance

The ATX–LPA axis is not only implicated in obesity, but may play an important role in the regulation of glucose homeostasis and insulin sensitivity. Subjecting ATX^+/−^ and FATX^−/−^ mice to an obesogenic diet results in improvements in systemic glucose tolerance and insulin resistance compared to the wild type [30,31]. Interestingly, however, overexpression of ATX in mice does not significantly alter glucose tolerance [75]. A single intraperitoneal injection of a supraphysiological dose (~1.4–1.5 mM) of LPA in male chow- and HFHS-fed C57Bl6 mice impairs glucose tolerance [15]. These acute systemic effects appear broadly mediated by LPA1 and LPA3, as pre-injection of a dual LPA1/3 antagonist, Ki16425, negates the LPA-mediated impairment in glucose tolerance [15]. Importantly, chronic treatment with Ki16425 also improves glucose and insulin tolerance in insulin resistant HFHS-fed mice [15]. On the contrary, intravenous administration of ~100–200 µM LPA in ICR or streptozotocin-diabetic mice lowers blood glucose and improves glucose tolerance [78]. The reasons for the discrepancies between these two studies are unclear, but may involve differences in LPA dose, vehicle, and/or mouse strain.

Studies in humans show that serum ATX levels correlate with several measures of glucose homeostasis and insulin sensitivity, including fasting glucose and insulin, glucose infusion rate (GIR), and homeostatic model assessment of insulin resistance (HOMA-IR) in overweight or obese nondiabetic individuals [66,68]. Additionally, serum ATX predicts measures of glucose homeostasis and insulin sensitivity in older humans [68]. In agreement with these studies examining ATX protein in serum, ATX mRNA levels are significantly higher in the intra-abdominal adipose tissue of massively obese women who exhibit impaired glucose tolerance or diabetes when compared to women with normal glucose tolerance [79]. Taken together, clinical evidence suggests that the ATX–LPA axis is positively associated with impaired glucose homeostasis and insulin resistance, and that ATX-LPA may serve as a therapeutic target and/or marker for obesity-related insulin resistance in humans.

While a relationship between ATX-LPA and systemic glucose homeostasis is well established (Table 1), the underlying mechanisms and effect of ATX-LPA signaling on tissue insulin function and metabolism are less well-understood. Improved glucose tolerance in HFHS-fed mice subjected to chronic (three weeks) administration of a LPA1/3 antagonist is associated with metabolic changes in multiple insulin sensitive tissues, including increased glycogen storage in the liver, glucose oxidation in skeletal muscle, and pancreatic islet mass [15]. Increased hepatic glycogen synthesis is paralleled by reduced mRNA expression of enzymes involved in gluconeogenesis, including glucose-6-phosphatase and phosphoenolpyruvate carboxykinase, in HFHS-fed mice treated with LPA1/3 antagonist [15]. In agreement with these findings, incubation of primary hepatocytes with LPA for 5-12 hr leads to the inhibition of insulin-stimulated glucokinase expression and glycogen synthesis, effects that are mediated primarily by LPA3 [69]. In 3T3-L1 adipocytes, a more chronic (16 hr) incubation with LPA impairs insulin signaling, as determined by reduced AKT phosphorylation [58]. However, a 24-h inhibition of ATX activity using PF-8380 does not alter insulin-stimulated AKT phosphorylation in insulin-sensitive or insulin-resistant 3T3-L1 adipocytes [55]. Few studies have examined the effect of very acute stimulation with LPA on the cellular insulin signaling pathway, with conflicting results: preincubation of primary rat hepatocytes with LPA for 15 min impairs insulin-stimulated AKT phosphorylation [69]; while a 10-min incubation with LPA promotes increased AKT phosphorylation, GLUT4 translocation to the plasma membrane, and 2-deoxyglucose uptake in 3T3-L1 and L6-GLUT4myc myotubes at baseline, although the effect of LPA on insulin-stimulated cells was not examined [78]. Taken together, most studies suggest that ATX-LPA-LPA1/3 signaling promotes glucose intolerance and impairs systemic insulin sensitivity and tissue insulin signaling. Future studies should clarify the precise molecular mechanisms by which the ATX-LPA pathway influences glucose homeostasis and insulin signaling in vivo and in vitro, and examine the role of individual LPA receptors in this process.

### Potential Mechanisms by which the ATX–LPA Axis Influences Insulin Resistance

Obesity is characterized by chronic low levels of systemic and tissue inflammation [80]. Landmark studies showed that pro-inflammatory factors, such as tumor necrosis factor α (TNFα), are elevated systemically and locally within the adipose tissue of murine models of obesity and diabetes, and contribute directly to obesity-induced insulin resistance [81]. TNFα neutralization improves peripheral insulin sensitivity in these models, demonstrating a key role of inflammation in the development of insulin resistance [81]. In addition to TNFα, several other pro-inflammatory cytokines are increased in obesity and contribute to the development and/or exacerbation of insulin resistance, including monocyte chemoattractant protein-1 (MCP1), interleukin-1 (IL-1), and IL-6 [82].

**Figure 3:**
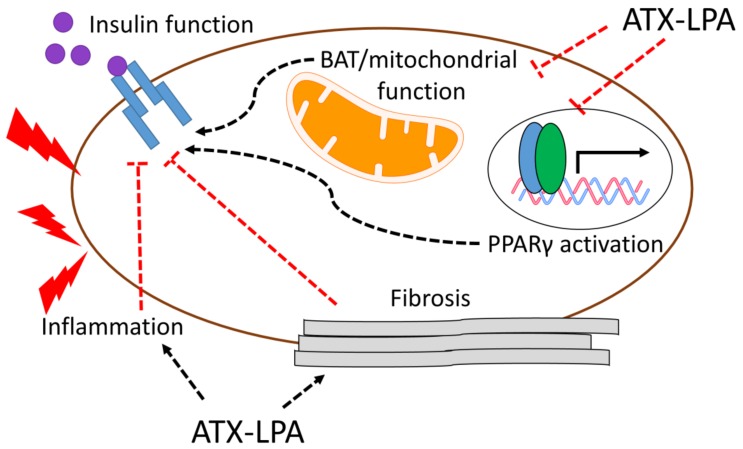
Potential mechanisms by which ATX-LPA signaling promotes insulin resistance and impaired glucose homeostasis. The ATX-LPA pathway may contribute to obesity-induced insulin resistance by stimulating inflammation and fibrosis and/or suppressing brown adipose tissue (BAT) and mitochondrial function, and PPARγ signaling.

Increased ATX-LPA signaling is linked to inflammation and inflammatory disorders including rheumatoid arthritis and hepatitis (Figure 3) [12,83]. Exposure of 3T3-L1 and 3T3-F442A adipocytes to the inflammatory cytokines IL6 and TNFα leads to the upregulation of ATX mRNA [58,79]. Similarly, inhibiting the pro-inflammatory transcription factor NFκB in 3T3-L1 adipocytes downregulates ATX mRNA [58]. ATX is not only stimulated by inflammation, but appears to enhance inflammation in a feed-forward mechanism. FATX^−/−^ mice show a significant decrease in adipose tissue and circulating levels of IL-6, TNFα, and MCP-1 [31]. Interestingly, overexpression of ATX driven by the α1-antitrypsin promoter does not systemically alter IL-6 and TNFα [75]. Although circulating ATX and LPA were elevated in this mouse model, adipose tissue levels of LPA were unchanged [75], suggesting that the ATX-LPA-induced stimulation of inflammatory cytokines is primarily due to enhanced ATX-LPA signaling in adipose tissue. Upregulation of pro-inflammatory cytokines in response to ATX-LPA pathway stimulation likely originates from immune cells. Coculture of 3T3-L1 preadipocytes and bone marrow-derived macrophages [BMDMs] increases levels of TNFα in BMDMs; these increases are abolished by ATX knockdown in preadipocytes [31]. Similarly, incubation of adipose tissue CD8^+^ T cells with recombinant ATX increases expression of CD44 and interferon-γ, which play a predominantly proinflammatory role [31]. Importantly, IL-6-mediated lipolysis and induction of systemic insulin resistance in HFD-fed mice require ATX and LPA1/3, since administration of Ki16425 for one week decreased plasma free fatty acids and improved glucose homeostasis [58]. Taken together, these studies suggest that local and systemic inflammation constitute an important mechanism by which the ATX-LPA pathway promotes insulin resistance.

Chronic inflammation can lead to the production of excessive connective tissue, giving rise to tissue fibrosis [84,85]. Fibrosis in adipose tissue is positively correlated with BMI and negatively correlated with insulin sensitivity in humans [86,87]. Increases in ATX-LPA-LPA1 signaling are linked to multiple fibrotic diseases, including idiopathic pulmonary fibrosis [11], chronic liver diseases [10], renal interstitial fibrosis [88], and scleroderma [89]. An unbiased, microarray-based approach in brown preadipocytes revealed that ATX-LPA signaling increases the expression of proteins involved in extracellular matrix remodeling [75]. Treatment of obese-diabetic *db*/*db* mice with Ki16425 for seven weeks improves systemic insulin sensitivity, which is associated with reduced adipose tissue fibrosis [77]. Exposure of human adipose tissue explants to LPA increases collagen 3 and the profibrotic cytokine transforming growth factor β (TBFβ), effects that are abolished upon coincubation with Ki16425 and are dependent on activation of hypoxia inducible factor 1α (HIF1α) [77]. Interestingly, adipose tissue of HFD-fed ATX^+/−^ mice does not show any significant changes in collagen 1a or 6a mRNA levels, indicating the absence of overt fibrosis [31]. Taken together, these studies suggest that the ATX-LPA pathway promotes fibrosis in severe cases of insulin resistance/diabetes (e.g., *db*/*db* mice), a mechanism by which ATX-LPA may further exacerbate impaired insulin function (Figure 3).

Altered energy homeostasis, signified by greater intake than expenditure of calories, is a hallmark of obesity and obesity-induced insulin resistance. BAT thermogenesis through respiration uncoupling plays a key role in regulating energy expenditure in rodents and humans [90,91,92,93,94]. Studies on humans demonstrate an inverse relationship between BAT activity and obesity/BMI [95,96,97]. In line with this notion, increasing BAT activity through cold acclimatization increases glucose disposal and improves insulin sensitivity [98,99]. Moreover, BAT transplants in the visceral cavity of mice improve glucose homeostasis, lower fat mass, and reverse diet-induced insulin resistance [100]. These studies demonstrate that BAT activity is a primary determinant of organismal energy expenditure. ATX-LPA signaling plays a key role in adipocyte proliferation and differentiation in both white and brown adipose tissue. Inhibition of ATX activity using HA155 or PF-8389 promotes the differentiation of primary BAT preadipocytes, concomitant with UCP1 upregulation [75]. Conversely, adding recombinant ATX or LPA directly to BAT preadipocytes inhibits their differentiation and decreases UCP1 and Prdm16, a master regulator of BAT differentiation [75]. Similarly, a microarray-based approach in brown preadipocytes revealed that ATX-LPA signaling downregulates proteins involved in mitochondrial function and energy metabolism [75]. Mice with ATX overexpression exhibit a reduction in inducible BAT, UCP1, and transcriptional regulators of mitochondrial biogenesis in white adipose tissue [75]. Interestingly, while these mice show increased diet-induced obesity, glucose homeostasis is unchanged [75]. In HFD-fed FATX^−/−^ mice, improved insulin sensitivity is associated with enhanced BAT activity and energy expenditure [31]. Morphologically, 70% of adipocytes from HFD-fed FATX^−/−^ mice show multiple lipid droplets, compared to 30% of adipocytes from HFD-fed WT controls, which is mirrored by increased mRNA expression of UCP1 and PGC1α, along with increased mitochondrial membrane potential in FATX^−/−^ mice [31]. Overall, these data suggest that ATX-LPA signaling inhibits BAT development and function, which may promote diet-induced insulin resistance (Figure 3). Future studies should explore how altered ATX-LPA signaling in other metabolically active tissues, including skeletal muscle, influences energetics and mitochondrial mass and function.

Peroxisome proliferator-activated receptor gamma (PPARγ) is a ligand-activated transcription factor that regulates various metabolic processes, including glucose and lipid homeostasis [101]. There are two major isoforms of PPARγ: PPARγ1, which is widely expressed; and PPARγ2, which is primarily expressed in adipose tissue [102]. A role for PPARγ in insulin resistance is evident in that several dominant negative PPARγ mutations are present in some patients with severe insulin resistance [103]. Thiazolidinediones (TZD), a class of drugs that activate PPARγ, are used clinically for their ability to act as insulin sensitizers [102]. The mechanisms by which PPARγ and TZDs promote insulin sensitivity are complex, multifactorial, and involve several tissues [104]. Interestingly, treatment of 3T3-L1 and 3T3-F442A adipocytes with the TZD rosiglitazone decreases ATX mRNA, protein content, and secreted ATX activity, suggesting that PPARγ inhibits ATX-LPA signaling [55,79]. The mechanism by which this occurs is unknown, but could involve a negative regulation of pro-inflammatory cytokines and transcription factors by PPARγ agonists [105,106,107]. Conversely, the ATX–LPA axis appears to reciprocally downregulate PPARγ signaling. FATX^−/−^ mice fed an obesogenic diet show increased mRNA levels of PPARγ and PPARγ sensitive genes (adiponectin, Glut-1, Glut-4, and leptin) in subcutaneous adipose tissue, and elevated levels of circulating adiponectin [30,31]. Notably, circulating adiponectin is inversely correlated with obesity and insulin resistance, and has insulin sensitizing effects on skeletal muscle and liver [108,109,110,111,112]. Inhibition of ATX activity in 3T3-L1 adipocytes results in increased protein levels of PPARγ, adiponectin, CD36, and Glut-4 at baseline, but is not able to restore levels of these proteins in insulin-resistant adipocytes [55]. Taken together, the ATX-LPA pathway may contribute to obesity-induced insulin resistance by impairing PPARγ expression and activity (Figure 3). The mechanism by which this occurs remains to be elucidated. It should also be clarified whether the reciprocal negative regulation of PPARγ and inflammatory cytokines and transcription factors involve the ATX–LPA axis.

## 5. The ATX–LPA Axis—A Potential Link between Obesity/Insulin Resistance and Cardiovascular Disease

Obesity and insulin resistance are well-established risk factors for the development of cardiovascular disease [113,114,115,116]. However, the underlying mechanisms are incompletely understood. Since ATX-LPA signaling is implicated in cardiovascular disease, particularly atherosclerosis [54,117], it is tempting to speculate that changes in the ATX-LPA pathway play an important role in promoting cardiovascular disease during obesity/insulin resistance. LPA levels are significantly higher in coronary arteries harboring atherosclerotic lesions when compared to the systemic arterial circulation, implicating LPA in the pathophysiology of acute coronary syndrome [118]. Histopathological studies of carotid endarterectomy specimens revealed that LPA is highly distributed in the lipid-rich core and in the proximal region of lesions, comprising of foam cells, lipid deposits, and connective tissue [119]. During atherogenesis, LPA levels in the lesion increase due to the dysregulation of LPA homeostasis [120]. In human atherosclerotic plaques, LPA contributes to increased platelet activation through stimulation of LPA1 and LPA3 [117]. Moreover, evidence from preclinical studies showed that the ATX–LPA signaling axis can aggravate the pathophysiologic events underlying atherosclerosis [54]. The major sources of LPA during atherogenesis are low-density lipoproteins (LDLs), although in the later stage of atherosclerotic progression, PLA_2_-mediated production of endogenous LPA may also contribute to LPA levels in the atherosclerotic lesion [121]. The increased accumulation of LPA in the atherosclerotic lesion favors the release of chemotactic proteins such as monocyte chemotactic protein 1 (MCP-1), leading to increased macrophage recruitment to the atherosclerotic plaque [120]. LPA can also trigger inflammation by inducing a wide range of proinflammatory cytokines, including IL-8, eotaxin, macrophage inflammatory protein-1β, and IL-1β, indicating that LPA induces sterile inflammation in the vessel wall [122]. In endothelial cells, the ATX-LPA signaling pathway promotes oxidized LDL-induced chemokine (C-X-C motif) ligand (CXCL) secretion, which accelerates the progression of atherosclerosis by promoting monocyte recruitment in the vessel wall [46]. Furthermore, LPA promotes endothelial dysfunction by reducing the expression of nitric oxide synthase, increasing oxidative stress; and stimulating the endothelial permeability, endothelial stress fiber formation, and subendothelial retention of LDL, thereby facilitating the initiation of atherosclerotic lesion formation [119,123]. The increased accumulation of LPA in the atherosclerotic lesion induces the activation of perivascular mast cells and release of tryptase, which contributes to plaque destabilization [120]. Since LPA features several atherogenic and thrombogenic properties in the atherosclerotic lesion, interventions reducing LPA availability and signaling could serve as an effective therapeutic strategy to improve plaque stability and reduce thrombogenic events [119].

Besides playing an important contributing role in atherosclerosis, LPA signaling is also implicated in other cardiovascular diseases. Studies in cardiomyocytes suggest that LPA has prohypertrophic effects, which are mediated by LPA3 signaling to AKT and ERK-NF-kB [124,125]. In agreement with these data, cardiac-specific knockout of LPP3 results in cardiomyocyte hypertrophy and myocardial dysfunction in mice [126]. This study suggests that maintenance of LPA homeostasis is critical for normal functioning of the myocardium [126]. Increased serum levels of LPA following acute myocardial infarction (AMI) are also linked to AMI-related pathophysiology [127]. Indeed, LPA receptor signaling promotes cardiomyocyte hypertrophy and left ventricular remodeling after myocardial infarction [127]. Interestingly, however, LPA preconditioning of immature rat hearts attenuates myocardial injury and improves cardiac function following ischemia–reperfusion [128]. A different study shows that LPA-treated human CD34^+^ cells induces recruitment of M2 macrophages and modulates the production of proinflammatory cytokines, protecting cardiac cells from ischemia-induced apoptosis [129]. A prosurvival role of LPA was also demonstrated in mesenchymal stem cells transplanted into the myocardium after infarct [130]. Recently, the ATX–LPA axis was implicated in calcific aortic valve disease [47]. LPA produced by ATX derived from lipoprotein (a) and valve interstitial cells triggers aortic valve inflammation and mineralization [47]. Taken together, these studies suggest that the ATX-LPA pathway plays an important role in the pathophysiology of cardiovascular diseases. Despite indications that LPA signaling is upregulated in the obese heart [16], it remains to be tested if and to what extent the ATX–LPA axis impacts cardiovascular disease associated with obesity, insulin resistance, and diabetes, and whether LPA signaling influences cardiomyopathy and heart failure under these conditions.

## 6. Concluding Remarks

Recent research has clearly implicated the ATX-LPA-LPA1-6 signaling axis in the development of metabolic disorders, including obesity, insulin resistance, and impaired glucose homeostasis, as well as cardiovascular disease. Targeting the ATX-LPA-LPA1-6 pathway holds therapeutic potential, as this signaling axis may promote the development of metabolic disorders through multiple mechanisms involving inflammation, fibrosis, and impaired mitochondrial function and PPARγ activation.

Indeed, LPA receptors have emerged as promising drug targets. At least three LPA receptor antagonists are in clinical trials [131], while many more LPA receptor modulators have been developed or are in development [132]. The LPA1 inhibitors BMS-986202/AM152 and BMS-986020 passed phase I and II clinical trials, respectively, for idiopathic pulmonary fibrosis; while the LPA1/3 inhibitor SAR 100842 passed a phase II clinical trial for systemic sclerosis [131]. Several additional compounds targeting mainly LPA1-3 were tested in preclinical studies for the treatment of dermal and kidney fibrosis, neuropathic pain, cancer, rheumatoid arthritis, hydrocephalus, and spinal and traumatic brain injury [131]. In addition, six specific ATX inhibitors underwent preclinical tests for the treatment of cancer, inflammation, asthma, idiopathic pulmonary fibrosis, and glaucoma [131]. The selective and potent ATX inhibitor GLPG1690 also recently passed a phase I clinical trial for idiopathic pulmonary fibrosis [133]. These studies demonstrate an immense interest of the research community and pharmaceutical industry in targeting the ATX-LPA receptor pathway for the development of pharmaceuticals for a variety of diseases, many of which are inflammatory in nature. Future studies should test the potential of ATX and LPA receptor modulators for the treatment of obesity- and diabetes-related metabolic disease and other comorbidities.

## Figures and Tables

**Table 1:** The influence of ATX-LPA signaling on adipocyte proliferation and differentiation, diet-induced obesity, insulin resistance (IR), and glucose intolerance (GI).

Effect of ATX and/or LPA on:				Models	Ref.
Preadipocyte proliferation	Preadipocyte differentiation	Diet-induced adiposity	Diet-induced IR/GI		
↑	↓	↑	↑	3T3-L1 (pre)adipocytes, primary murine preadipocytes, ATX^+/−^ mice, FATX^−/−^ mice, fat-specific ATX-overexpressing mice	[31]
↑	n.d.	n.d.	n.d.	3T3-F442A preadipocytes, NIH-3T3 fibroblasts	[71,72]
↑	↓	n.d.	n.d.	3T3-L1 preadipocytes, DFAT-P preadipocytes	[73]
↔	↓	↑	↔	Primary murine brown preadipocytes, ATX-overexpressing mice	[75]
n.d.	↓	↓	n.d.	3T3-F442A preadipocytes, SGBS preadipocytes, LPA1-KO mice, primary murine pre-adipocytes	[76]
n.d.	n.d.	↓	↑	FATX^−/−^ mice	[30]
n.d.	n.d.	↔	↑	Chow-fed *db*/*db* mice treated with LPA1/3 antagonist (Ki16425)	[77]
n.d.	n.d.	n.d.	↑	Chow- and HFHS-fed WT mice treated with Ki16425	[15]
n.d.	n.d.	↔	↑	3T3-L1 adipocytes, chow- and high-fat diet-fed WT mice treated with Ki16425	[58]
n.d.	n.d.	n.d.	↔	3T3-L1 adipocytes treated with ATX inhibitor (PF-8380)	[55]

↑, increased effect; ↓, decreased effect; ↔, no significant difference was observed; n.d., not determined; FATX, fat-specific autotaxin-knockout; DFAT-P, porcine dedifferentiated fat cells; SGBS, Simpson-Golabi-Behmel Syndrome; WT, wild type; KO, knockout.
